# Supplementary material for: An optofluidic antenna for enhancing the sensitivity of single-emitter measurements
Source: Nat Commun. 2024 Mar 21;15:2545. doi: 10.1038/s41467-024-46730-w (PMC10957926; doi:10.1038/s41467-024-46730-w)
Supplement: Supplementary file 1 — Supplementary Information [file 41467_2024_46730_MOESM1_ESM.pdf]

An optofluidic antenna for enhancing the sensitivity of single-emitter measurements.
Supplementary Information

Luis Morales-Inostroza[1,2,3], Julian Folz[4], Ralf Kühnemuth[4], Suren Felekyan[4], Franz-Ferdinand Wieser[1,2,3], Claus A.M. Seidel[4], Stephan Götzinger[1,3,5] and Vahid Sandoghdar[1,3]

[1]Max Planck Institute for the Science of Light, 91058 Erlangen, Germany
[2]Max-Planck-Zentrum für Physik und Medizin, 91058 Erlangen, Germany
[3]Department of Physics, Friedrich-Alexander-Universität Erlangen-Nürnberg, 91058 Erlangen, Germany
[4]Chair for Molecular Physical Chemistry, Heinrich Heine University Düsseldorf, 40225 Düsseldorf, Germany
[5]Erlangen Graduate School in Advanced Optical Technologies (SAOT), Friedrich-Alexander-Universität Erlangen-Nürnberg, D-91052 Erlangen, Germany

March 5, 2024

Contents

Supplementary Note 1	Experimental assembly of the optofluidic antenna	2
Supplementary Note 2	Fluorescence correlation spectroscopy	3
Supplementary Note 3	Calculation of the molecular detection efficiency (MDE)	4
Supplementary Note 4	Diffusion of differently charged dye molecules inside the OFA	6
Supplementary Note 5	Distribution of the excitation light in the antenna	6
Supplementary Note 6	Photon collection enhancement at the water-air interface	8
Supplementary Note 7	HJ - filtered FCS	10
Supplementary Note 8	Holliday Junction - sample	11
Supplementary Note 9	Holliday Junction - Photon Distribution Analysis (PDA)	12
Supplementary Note 10	HJ - FRET efficiency trajectories	12
Supplementary Note 11	Multiparameter Fluorescence Detection (MFD)	15
Supplementary Note 12	Monte-Carlo simulations of the molecular diffusion	15
Supplementary Note 13	Holliday Junction - sm-MFD	16

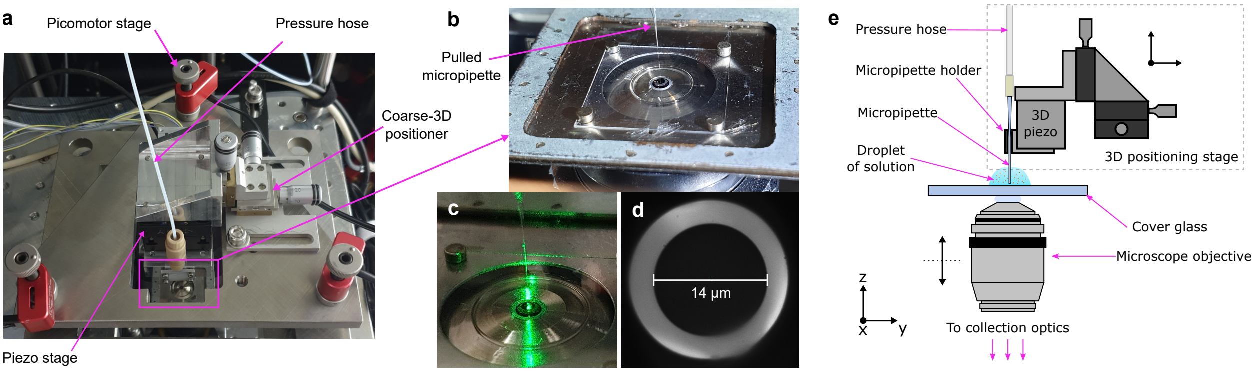

Supplementary Fig. 1: **Macroscopic system for assembling the optofluidic antenna. a,** Purpose-built sample holder for positioning the micropipette and aligning the micropipette end with respect to the cover glass. **b,** Micropipette aligned to the optical axis of the microscope objective and close to the cover glass. **c,** Zoom into the micropipette-cover glass arrangement. Scattering of the laser used in wide-field illumination mode allows clearer visualization of the micropipette. **d,** Image of the micropipette end captured with the microscope objective. The micropipette is surrounded by air. **e,** Sketch of the experimental arrangement.

Supplementary Note 1: Experimental assembly of the optofluidic antenna

Supplementary Fig. 1 **a** depicts the arrangement used for positioning the micropipette. Here, a 3-axis actuator (mini-Martock) is utilized for coarse alignment (micrometer precision) of the micropipette relative to the optical axis of the microscope objective. Moreover, the micropipette is mounted on a 3-axis piezo actuator (Physik Instrumente) mechanically coupled to the translations stage to control the distance between the micropipette and the cover glass with nanometer precision. Additionally, precision piezoelectric screws (Picomotor, New Focus) are implemented on the sample holder base plate for correcting the relative angle between the flat end of the micropipette and the cover glass.

A PolyTetraFluoroEthylene (PTFE) adapter is used in combination with a plastic tube that is connected to the non-tapered end of the micropipette. The other end of the tube is connected to a syringe (Setonic). Tuning the syringe plunger allows controlling the pressure inside the micropipette and, thus, the shape of the water meniscus.

Supplementary Fig. 1 **b** shows the micropipette when it is close to the cover glass. At this point, the micropipette is roughly aligned relative to the optical axis of the microscope objective. We image light scattered off the pipette end for visual guidance, facilitating the positioning of the micropipette in the objective field of view. Supplementary Fig. 1 **c** exhibits an image of the cover glass and the micropipette where the scattering of laser light, sent through the microscope objective, is visible. Supplementary Fig. 1 **d** displays an image of the micropipette end viewed from the microscope objective. In this case, the micropipette was surrounded by air. Additionally, a simplified sketch of the experimental setup is shown in Supplementary Fig. 1 **e**.

After the coarse alignment described above is completed, the gap between the cover glass and the micropipette is filled by adding a blank buffer solution next to the micropipette (see sketch in Supplementary Fig. 1 **e**). The fine alignment of the angle between the micropipette end and the cover glass is adjusted by monitoring the interference fringes formed in the thin water layer. During this procedure, the OFA is illuminated with laser light (532 nm) in wide-field configuration.

Supplementary Fig. 2 **a-d** shows the interference fringes formed as the angle between the micropipette end and the cover glass is corrected. When the micropipette end is positioned parallel to the cover glass, interference fringes have ideally vanished. Once the angle between the micropipette end and the cover glass is corrected, the thickness of the water layer can be adjusted via white light interferometry. The OFA is illuminated with a broad white light source. Supplementary Fig. 2**e** shows interference patterns for several

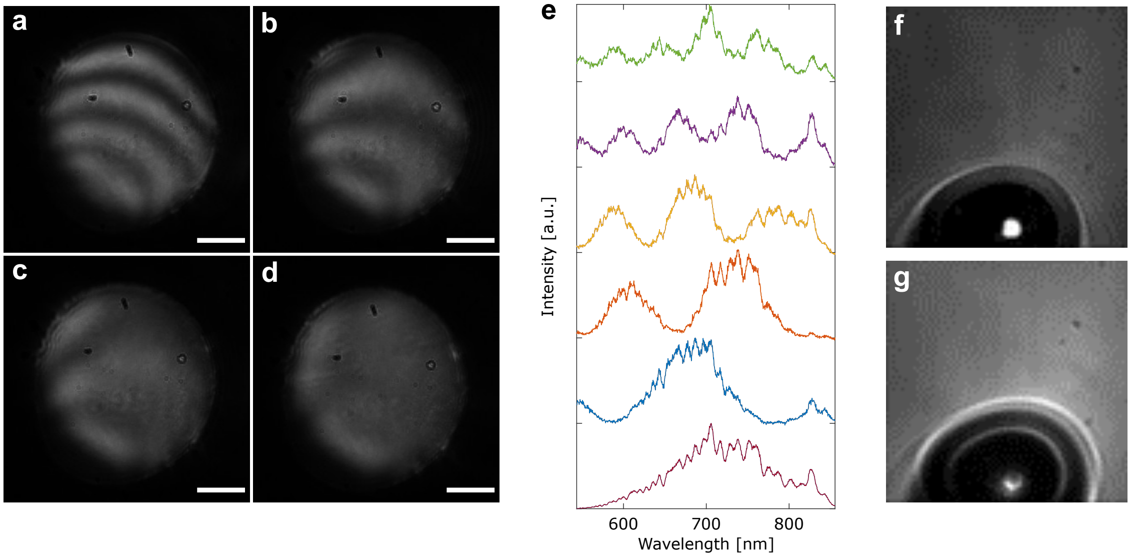

Supplementary Fig. 2: **Micropipette alignment. a-d,** Spatial interference patterns of the laser light, observed as the angle between the micropipette relative to the cover glass is corrected. The scale bar corresponds to 4μm. **e,** Interference pattern observed in the spectrometer due to white-light interference in the thin water layer formed between the water-air interface and the water-cover glass interface. The different spectra correspond to thicknesses of the water layer from top to bottom of, 6.0 μm, 3.5 μm, 1.5 μm, 1.0 μm, 0.5 μm and 0.3 μm. The fast modulation on top of the spectrum is an artifact produced by the optical setup. **f-g,** Images of the micropipette entering the field of view in two independent experiments.

thicknesses of the water layer. These interference patterns are recorded with a spectrometer implemented in the detection path. As the water layer thickness is reduced, the period of the white light interference is increased (see the figure caption for details). A similar procedure could also be implemented using a monochromatic light source such as an LED.

We note that we do not observe any change in the shape of the water meniscus within the duration of the measurements, which typically take several hours (as long as the macroscopic drop of solution next to the micropipette is not evaporated). Therefore, we can safely assume no significant evaporation of the solution via the capillary. This process is also unlikely to influence the meniscus shape since the volume of air in the thin and long capillary is small and, thus, quickly saturates above the liquid surface.

We also want to stress that the OFA can be assembled rather fast due to the reproducibility of the positioning system depicted in Supplementary Fig. 2**a**. A simple flashlight from the top allows for the assembling of the OFA by observing the shadow of the micropipette. Supplementary Figs. 2 **f** and 2 **g** show two images of independent experiments where a micropipette enters the field of view. Next, the micropipette is positioned at the center of the field of view (see Supplementary Fig. 1 **d**).). Moreover, at this point, the micropipette end and the cover glass are brought in contact. This step is visually monitored by looking at the displacement in the z-direction of the focus spot formed in the center of the micropipette. Afterward, the distance between the micropipette and the cover glass is tuned using a piezo actuator to displace the micropipette in the z-direction.

Supplementary Note 2: Fluorescence correlation spectroscopy

Fluorescence correlation analysis was performed in different scenarios. In the case of free dye molecules (i.e., not attached to a biomolecule), the fluorescence signal corresponded to 2-4 molecules in the observation volume. At the single-molecule (sm) level, between 2-5 bursts per second were registered, yielding an observation volume occupation ratio of roughly 1:1000. Moreover, to resolve dynamic events, the signal was filtered using lifetime information (see Ref. [1]). Fits of the correlation curves for the free dye series were

done using a correlation function including the triplet state of the dye and its translational diffusion in a 3-dimensional Gaussian-shaped volume as follows [2],

$$G(t_c) = b_0 + \frac{1}{N_{\text{bright}}} \left[1 + \frac{t_c}{t_d}\right]^{-1} \left[1 + \frac{t_c}{t_d(\frac{z_0}{\omega_0})^2}\right]^{-1/2} \left[1 - |A| + |A| e^{-t_c/t_A}\right]. \tag{1}$$

Here, b_0 denotes the offset, N_{bright} is the number of bright particles in the focus, t_d represents the diffusion time, A signifies the amplitude for the triplet state, and t_A is the time constant of the triplet state. For the free dye series, the brightness of a molecule was estimated using the number of molecules in the bright state as,

$$N_{\text{bright}} = N \cdot (1 - A), \tag{2}$$

and therefore the brightness Q of a molecule is given by

$$Q = \frac{F_{D|D}}{N} = \frac{F_{D|D}}{N_{\text{bright}}} \cdot (1 - A), \tag{3}$$

whereby $F_{D|D}$ signifies the background-corrected fluorescence of the donor after excitation. Since increasing the excitation intensity leads to saturation, we fitted the brightness as a function of the excitation intensity using a saturation curve given by (see Fig. 2**b** in the main text) [3],

$$Q(I_{\text{exc}}) = Q_{\text{sat}} \frac{I_0/I_\sigma}{1 + I_0/I_\sigma}, \tag{4}$$

where I_σ corresponds to the cross-section excitation power and is fitted globally. Q_{sat} is the saturated brightness of a molecule at the maximum I_{exc}.

We note in passing that when using the OFA, high values of z_0/w_0 were fitted, with an average value of approximately 13. This figure suggests a 2D diffusion behavior of the observed particles, whereas a measured value for z_0/w_0 of approximately 4 was found for particles in an open solution, which reports a normal 3D diffusion.

Supplementary Note 3: Calculation of the molecular detection efficiency (MDE)

Detected fluorescence counts in a confocal microscope are characterized by the focus of the excitation beam, the point-spread function (PSF) at the image plane and the transmission function of the pinhole in the detection path [5]. The excitation can be approximated as a Gaussian beam with intensity distribution

$$\begin{aligned} I(r,z) &= \frac{w_0^2 I_0}{w^2(z)} \cdot e^{-\frac{2r^2}{w^2(z)}} \\ w^2(z) &= w_0^2 + \left(\frac{\lambda}{n\pi w_0}\right)^2 z^2 \end{aligned} \tag{5}$$

where I_0 is a normalization constant. In the simulations, we set the lateral dimension of the beam waist w_0 to 1000 μm, the excitation wavelength to $\lambda_{exc} = 532$ nm and the refractive index of the medium to n=1.33 in order to mimic the experimental conditions. While the PSF is often approximated as that of a point-like source in a homogeneous medium, in the case of OFA one should account for the vectorial nature of radiation from a dipole in the stratified medium. We calculate the PSF for horizontally and vertically oriented dipoles using a semi-analytical approach presented by Mortensen et al. [6] for different thicknesses of the water layer ranging from 0.1 μm to 5 μm, a numerical aperture NA=1.46 and an emission wavelength λ_{em}=550 nm. Supplementary Fig. 3**a** displays an example of the lateral cross-sections in a 1 μm water layer.

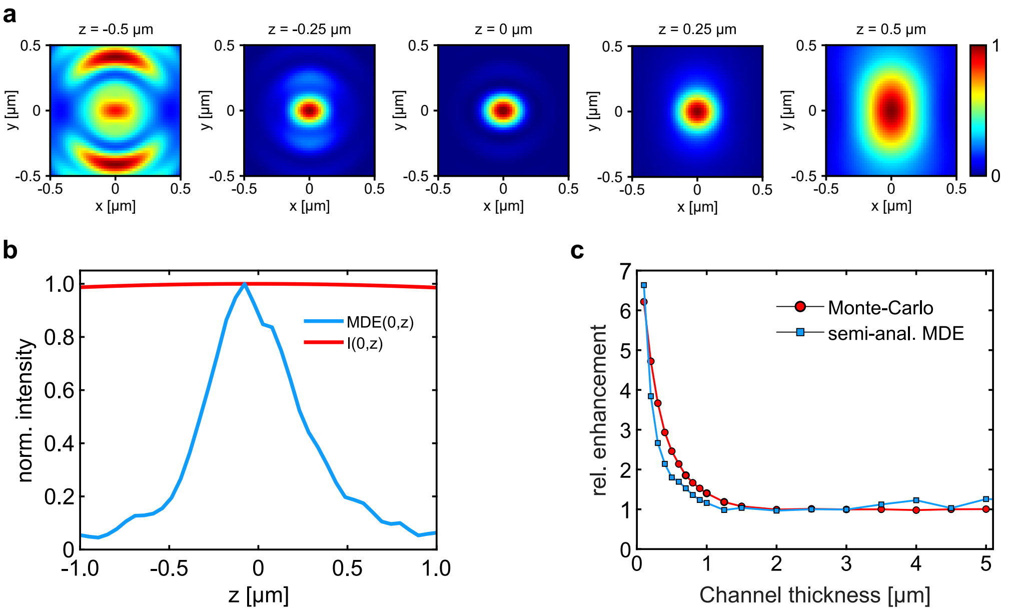

Supplementary Fig. 3: **Relative enhancement of photon collection in the OFA. a.** Cross-section of the PSF of a horizontally-oriented (x-axis) dipole inside a 1 μm water layer bounded by cover-glass (n=1.5) and air (n=1). **b.** Molecular detection efficiency of the OFA (blue) and the excitation profile (red). **c.** Relative enhancement of the position-averaged MDE for small water layer thicknesses. The blue line represents a semi-analytical calculation based on vectorial diffraction, and the red line is a Monte-Carlo simulation of a diffusing molecule inside water layers with varying thickness for the same parameters of the observation volume.

The effect of the pinhole is to limit the transmission of the signal in the image plane since the fraction of light that passes depends on the position of the image with respect to it. Thus, we define the molecular detection efficiency (MDE) as

$$\mathrm{MDE}(r,z) = I(r,z) \cdot \int \mathrm{PSF}(r,r',z) \cdot T(r') \tag{6}$$

whereby $T(r)$ is given by

$$T(r) = \begin{cases} 1 & r < 0.5\mu m \\ 0 & r > 0.5\mu m \end{cases} \tag{7}$$

is the transmission function of the pinhole. The quantity MDE can be computed for horizontal and vertical dipole orientations (H, V), respectively. Supplementary Fig. 3**b** shows the axial profile along r=0 together with the excitation profile. The orientation-averaged MDE is then given by

$$\mathrm{MDE}(r,z) = \frac{2}{3}\mathrm{MDE}_H + \frac{1}{3}\mathrm{MDE}_V \tag{8}$$

The relative enhancement of the position-averaged MDE inside the OFA compared to an open solution is shown in Supplementary Fig. 3 **c** and compared with a Monte-Carlo simulation of diffusion through the observation volume. For the Monte-Carlo simulations, the PSF of the emitter is approximated by a spherical wavefront.

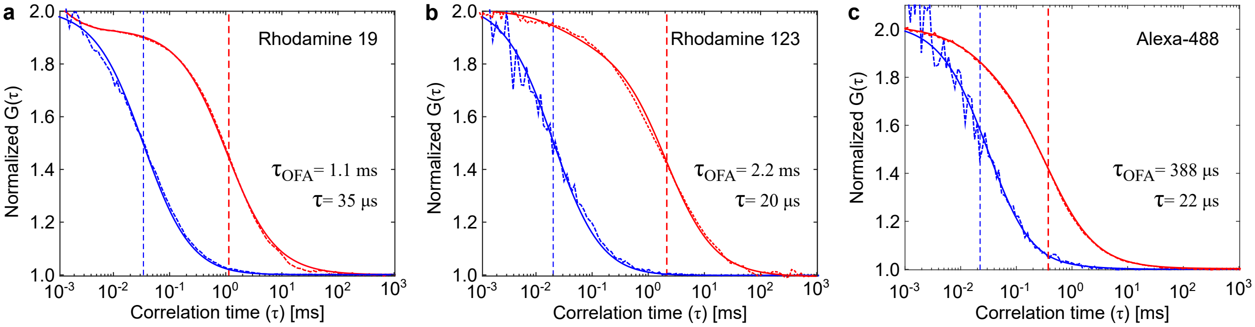

Supplementary Fig. 4: **Charge-dependent diffusion enhancement.** Normalized cross-correlation (dotted red, blue curves) and corresponding fit (continuous red, blue curves) obtained for free dye molecules in the optofluidic antenna (red curves) and in bulk solution (blue curves). The diffusion time in an open solution (τ) and inside the OFA (τ_{OFA}) extracted from the fits are shown in the legend and also indicated with vertically dotted blue (τ) and red (τ_{OFA}) lines. All measurements were performed at picomolar concentration and with 10 μW excitation power using a 532 nm (**a**) or 488 nm (**b**, **c**) laser.

Supplementary Note 4: Diffusion of differently charged dye molecules inside the OFA

The nature of the interaction between analytes and the water-air interface remains unknown. To gain more experimental insight into this issue, we examined the diffusion of Rhodamine-19 (positively charged), Rhodamine-123 (positively charged), and Alexa-488 (negatively charged). For this experiment, the antenna geometry was fixed to a water layer thickness of 0.5 μm. Depending on the specific dye, the excitation wavelength was chosen to be 488 nm (Toptica iBEAM SMART 488) or 532 nm (COHERENT-Sapphire 532-200). In both cases, the excitation power was set to 10 μW to avoid photodamage. Supplementary Fig. 4 shows the normalized autocorrelation corresponding to the different dyes mentioned above with (red) and without (blue) OFA. Here, positively-charged molecules Rhodamine-19 and Rhodamine-123 show an increase in the diffusion time by a factor of 110 and 31, respectively, whereas negatively charged dye molecules Rhodamine-110 (see Fig.2 **b** in the main text) and Alexa-488 exhibit a considerably smaller enhancement of 7.5 fold and 17 fold in the diffusion time, respectively. We remark that in this experiment, the lateral extension of the observation volume has been reduced to $w_0 = 400$ nm to confirm that the enhanced diffusion time is not an artifact produced by the size of the observation volume.

Supplementary Note 5: Distribution of the excitation light in the antenna

We used the "Thin lens" package from "Lumerical Inc. V.8.20.1731" to solve the full vectorial model of Maxwell's equations inside the OFA dielectric structure. As an initial input, we chose a Gaussian beam polarized along the x-axis. Moreover, a lens with a NA of 1.46 or 0.4 was used to simulate light focused to tight and large confocal spots, respectively, whereby the light source was set to fill the extension of the lens. In either case, the beam was mapped using 300 plane waves, and the wavelength was set to 532 nm. Furthermore, the distance of the lens from the focal plane was 1.6 μm in the case of a lens with NA=1.46, and 8 μm in the case of a lens with NA=0.4. For high precision determination of the confocal volume, a mesh grid with 5 nm resolution was applied in the region corresponding to the water layer. We note that in all numerical simulations, the water-air interface is assumed to be flat in the region of the observation volume. This is justified because the height of the water layer remains constant over the extension of the entire laser spot to within a few nanometers.

First, we examine the scenario where the laser beam is tightly focused inside the water layer. Supplementary Figs. 5 **a** and **b** show the intensity distribution for the case of tight focusing in an antenna formed by a

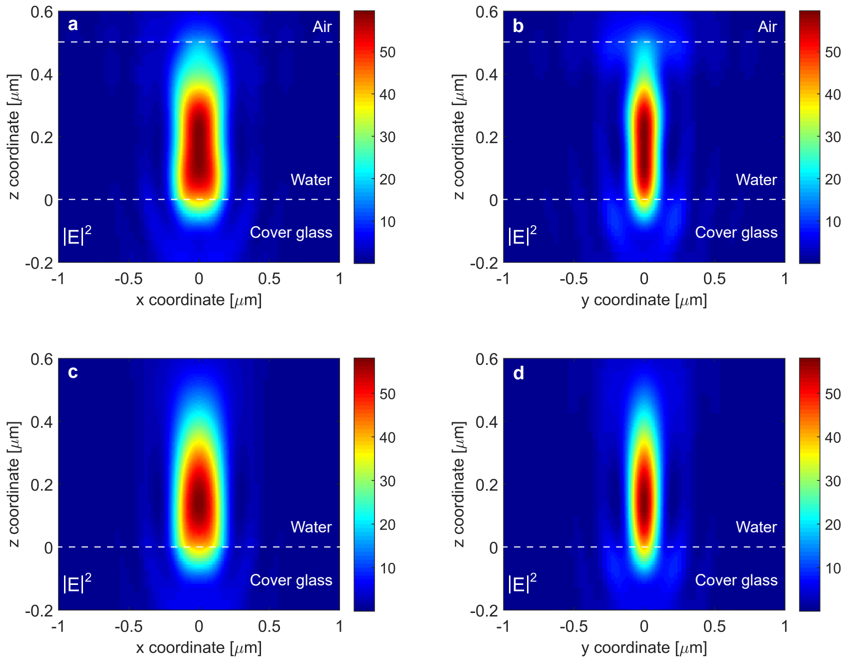

Supplementary Fig. 5: **Intensity profile of the excitation volume inside the OFA for NA=1.46.** Intensity distribution of the confocal spot inside the optofluidic antenna at the x/z ($y = 0$ nm) plane (**a**) and at the y/z ($x = 0$ nm) plane (**b**). The dashed white lines indicates the interfaces of the materials constituting the OFA. Intensity distribution of the confocal spot passing through the glass-water interface at the x/z ($y = 0$ nm) plane (**c**) and at the y/z ($x = 0$ nm) plane (**d**). The horizontal dashed white line indicates the cover glass-water interface.

cover glass (n =1.517), 500 nm of water (n =1.33), and air (n =1.0). For this choice of water layer thickness, the electric field distribution produced by the excitation laser inside the OFA is only slightly modified as compared to the ideal Gaussian shape. Moreover, the volume confined up to I_0/e^2 (width of a beam with maximum intensity I_0) is only 5% smaller, and the intensity maximum is 3% higher compared to the case of the confocal volume at the glass-water interface. Supplementary Figs. 5 **c** and **d** show the case of a focused laser beam passing through a glass-water interface, i.e., in the absence of an OFA. The intensity distribution of the electric field is shown in **c** and **d** at the x/z ($y = 0$ nm) and y/z ($x = 0$ nm) planes, respectively. We note that the difference between the beam width at the x-z and y-z planes is due to the linear polarization of the input beam in combination with the high NA of the lens used to focus the light [4].

Next, we consider the case of a large confocal spot passing through the OFA. Supplementary Figs. 6 **a** and **b** show the intensity distribution obtained for focusing with a lens with NA=0.4. and antenna geometry consisting of a cover-glass (n =1.517), 500 nm of water (n =1.33), and air (n =1.0). In this scenario, the laser beam can be considered to a good approximation as a plane wave. Indeed, in the simulation we observe a modulation of the light along the z-axis produced by the interference of incident and reflected waves at the cover glass-water interface, as well as at the water-air interface. The visibility of the interference reaches 0.24 inside the water layer (see Supplementary Fig. 6 **c**). Supplementary Figs. 6 **d** and **e** represent the intensity distribution obtained when the light passes through the water-air interface at the x/z ($y = 0$ nm) and y/z ($x = 0$ nm) planes, respectively. Finally, Supplementary Fig. 6 **f** shows the intensity modulation along the z-direction when focusing the light at the glass-water interface. Here, we can see that in the absence of the water-air interface, the light intensity modulation occurs only in the glass substrate, and the intensity distribution in water is that of bulk solution. In the case of a large observation volume, the main effect of the water-air interface is to restrict the analytes' diffusion. Depending on the thickness of the water layer, the observation volume can be reduced by up to tenfold (for a 500 nm water channel thickness) relative to the case of diffusion in bulk solution. We note that the asymmetry in the x-z and y-z planes produced by

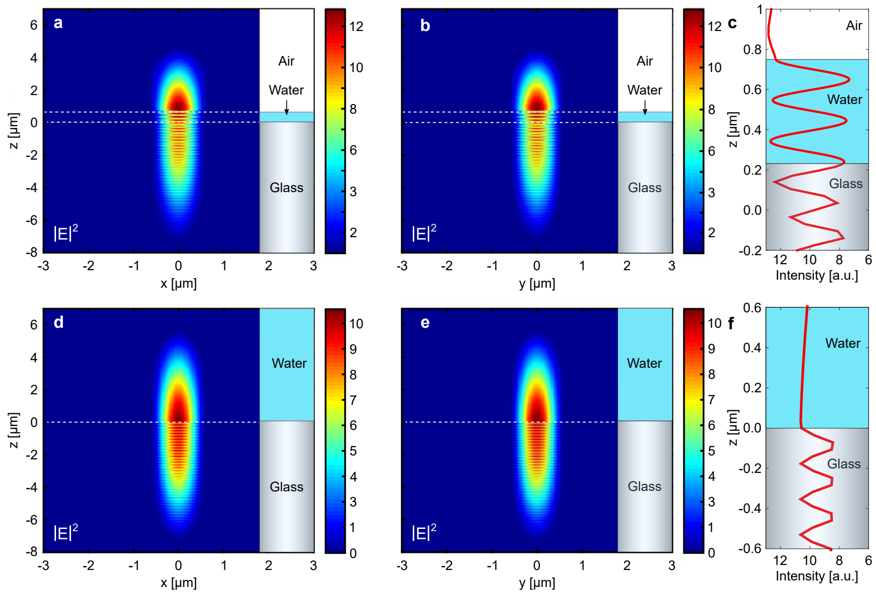

Supplementary Fig. 6: **Intensity profile of the excitation volume inside the OFA for NA=0.4.** Intensity distribution of the confocal spot inside the optofluidic antenna at the x/z ($y = 0$ nm) plane (**a**) and at the y/z ($x = 0$ nm) plane (**b**). The dashed white lines indicates the interfaces of the materials constituting the optofluidic antenna. **c,** Intensity distribution of the confocal spot inside the OFA along the z-direction for $x = y = 0$. Intensity distribution of the confocal spot at the glass-water interface. Supplementary Figs. **d** and **e** corresponds to the planes x/z ($y = 0$ nm) and y/z ($x = 0$ nm), respectively. The dashed white line indicates the glass-water interface. **f,** Intensity distribution of the confocal spot at the glass-water interface along the z-direction for $x = y = 0$.

the linear polarization of the input beam is no longer visible in the case of focusing with a low NA lens.

Supplementary Note 6: Photon collection enhancement at the water-air interface

Here, we show that a simple water-air interface also enhances the photon collection efficiency as compared to the case of conventional open solution. Again, several effects can contribute.

Supplementary Fig. 7 **a** shows a cut through the radiation pattern calculated for a dipole-like emitter in open solution. Here, we have assumed a fast randomly changing dipole orientation such that the obtained emission pattern is symmetric along the azimuth (ϕ). Subwavelength vicinity of the interface can substantially modify the radiation pattern and thus direct the emission towards the collection optics. Supplementary Figs. 7 **b** and **c** show the radiation patterns obtained from an emitter positioned at 1 nm, and 100 nm from the water-air interface, respectively. For comparison, we also include the emission pattern of an emitter inside the OFA in Supplementary Fig. 7 **d**, where the water layer thickness is set to 500 nm, and the emitter position is 250 nm from the water-air interface. Supplementary Fig. 7 **e** shows the radiated power in the y-z plane as a function of the collection angle for the different cases shown from **a** to **d**. Additionally, we have included the distribution of the radiated power of an emitter located at 1000 nm from the water-air interface (gray curve). Supplementary Fig. 7 **f** displays the corresponding photon collection efficiencies in the y-z plane again as a function of the collection angle (labeled with the same color code as in (e)). A simple water-air interface can efficiently channel more than 75% of the total radiated power into angles covered by a collection optics with NA=1.46. This corresponds to about 85% higher photon collection as compared to the case of an emitter in open solution. This enhancement can be exploited as long as the emitter is located close to the water-air interface. Furthermore, the collection of light radiated by an emitter close to the air-water

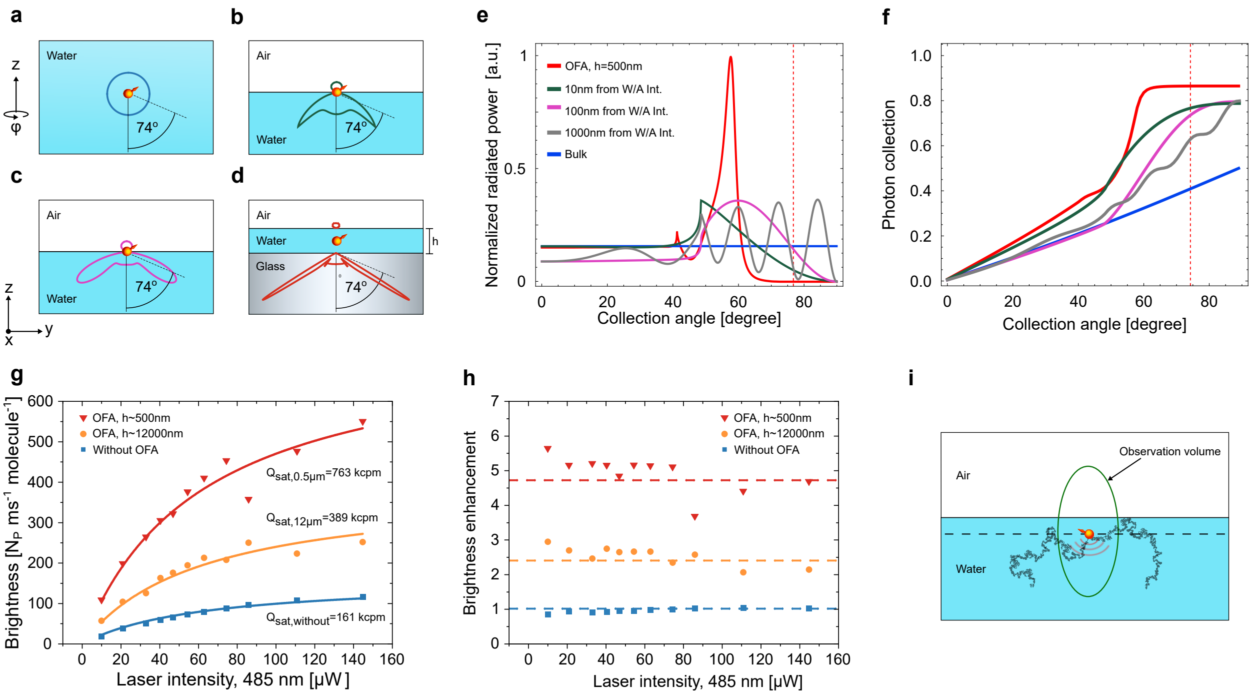

Supplementary Fig. 7: **Orientation averaged emitter in different dielectric structures.** Cut along the y-z plane to display the radiation pattern in an open solution (**a,**), at 1 nm from the water-air interface (**b,**), at 100 nm from the water-air interface (**c,**), and in the center of an OFA (**d,**) with h=500 nm. **e,** Radiated power in the y-z plane for the arrangements shown in **a** to **d**, and for the case of an emitter at 1000 nm from the water-air interface as a function of the collection angle. The red vertical dotted line indicates the maximum collection angle of a lens with 1.46 NA. **f,** Calculated photon collection efficiency in the y-z plane. The red vertical dotted line indicates again the maximum collection angle of a lens with 1.46 NA. **g,** Power-dependent brightness saturation obtained in confocal measurements of freely diffusing Rhodamin110 molecules obtained via FCS (see main text for details). The blue and red curves correspond to the case of analytes diffusing in an open solution and inside the OFA, respectively. The orange curve corresponds to the case of an ODA with h=12μm, where the center of the observation volume coincides with the water/air interface. **h,** Experimental brightness enhancement obtained for Rhodamine-110 dye molecules diffusing in open solution (blue data points), inside an OFA with h=500 nm (red data points), and inside an OFA with h=12000 nm (orange data points). The horizontal dashed lines indicate the average brightness enhancement in each case. In the case of an OFA with h=12000 nm, the focal plane of the lens used for collection is set just below the water-air interface to simulate a scenario where an emitter is in the close vicinity of a water-air interface (Supplementary Figs. **b** and **c**). **i,** Sketch of a confocal-based measurement at the water-air interface. The black dashed horizontal line indicates the focal plane.

interface can be further enhanced due to refraction at the glass-water interface.

Supplementary Fig. 7 **g** shows the brightness per molecule as a function of the excitation power obtained experimentally for different experimental arrangements (curves red and blue are also shown in the main text). In Supplementary Fig. 7 **h**, we show a comparison of the experimentally obtained brightness enhancement as a function of the excitation power for the case of Rhodamine-110 molecules diffusing in an open solution (blue data points), inside an OFA with h=500 nm (red symbols), and inside an OFA with $h = 12\,\mu m$ (orange data points). A 2.4-fold enhancement of brightness is evident in the latter case. As shown in Supplementary Fig. 7 **i**, the focal plane was set slightly below the water-air interface. In this case, the MDE is also enhanced since the observation volume is more confined as compared to the case of an open solution (see discussion in the main text around Fig. 2 **a**).

The arrangement illustrated in Supplementary Fig. 7 **i** is readily available to any conventional FCS-type measurement setup and offers a valuable means for achieving some of the advantages of a proper OFA. A

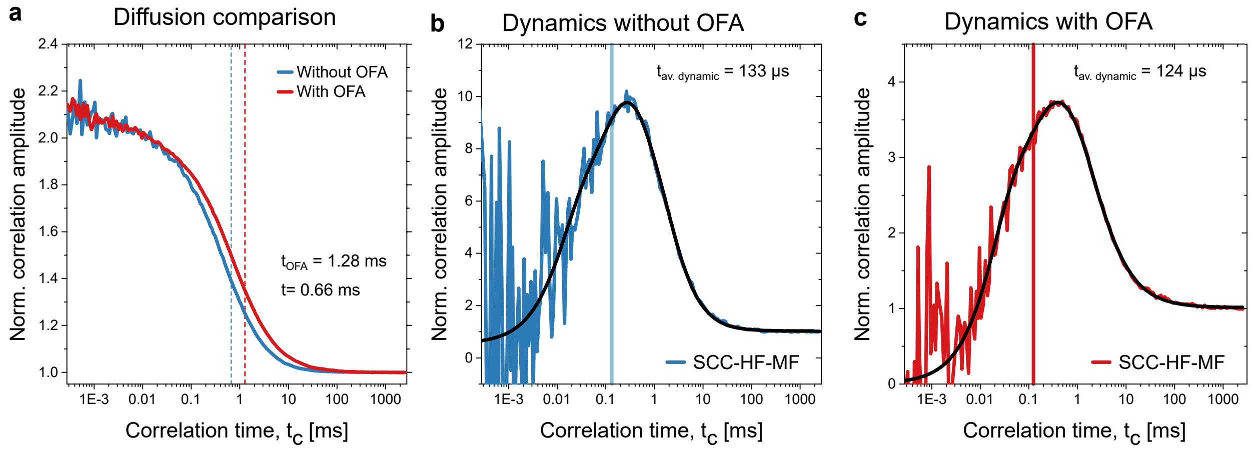

Supplementary Fig. 8: **HJ dynamics analysis using species-filtered Fluorescence Correlation Spectroscopy. a,** Lifetime filtered, medium FRET (MF) species auto-correlation function of the Holliday junction D(a)A(b) without Mg^{2+} with (red curve) and without (blue curve) the OFA. The vertical blue and red dashed lines indicates the diffusion time obtained from the corresponding fit, also shown numerically as inset. Supplementary Figs. **b** and **c** correspond to the High FRET (HF) and medium FRET (MF) species cross correlation of the HJ without (blue curve) and with (red curve) antenna. The vertical blue and red lines indicate the average relaxation time also show as inset.

quantitative study of this case is beyond the scope of our current work and is left for future studies.

Supplementary Note 7: HJ - filtered FCS

Another approach to resolving fast dynamics between conformations is filtered Fluorescence Correlation Spectroscopy (fFCS) based on Ref. [1]. Here, FRET species are separated using a lifetime-based filter. Selecting the HF and MF species of the HJ via burst selection leads to a distribution of short lifetimes for the HF state and longer lifetimes for the MF state. Moreover, applying these filters to the correlation analysis of all selected FRET bursts (a mixture of MF and HF) leads to a correlation curve between the FRET states. Furthermore, the correlation analysis provides information about the relaxation time between the HF and MF states in the form of an anti-correlation term.

To analyze the experimental data, we consider a global-fit approach using species-auto-correlation (SAC) and species-cross-correlation (SCC) curves. The fit of the SAC was done using equation 1 and for the fitting of the SCC we used the following equation,

$$G(t_c) = b_0 + \frac{1}{N_{bright}} \left[1 + \frac{t_c}{t_d}\right]^{-1} \left[1 + \frac{t_c}{t_d(\frac{z_0}{\omega_0})^2}\right]^{-1/2} \left[1 - |A| + |A| \cdot e^{-t_c/t_A}\right] \times \quad (9)$$

$$\left[1 - |AB| \cdot \left(|B|e^{-t_c/t_B} + |C|e^{-t_c/t_C} + (1 - |B| - |C|) \cdot e^{-t_c/t_D}\right)\right], \quad (10)$$

where AB corresponds to the total amplitude of all anti-correlation terms, and B, C, and $(1 - B - C)$ are the individual amplitudes of the different species. Analysis of the SAC shows that the diffusion time τ_d is almost three times higher using the OFA (see Supplementary Fig. 8**a**). On the other hand, the average dynamic relaxation time $t_{\mathrm{av,dyn}} \approx 100\mu s$ show no significant change when measuring the HJ inside the OFA compared to an extended solution (see Supplementary Figs. 8**b** and **c**). The combination of the MFD and fFCS analysis ultimately demonstrates that the use of the OFA affects neither the conformational states of the HJ nor the internal dynamic exchange rate between them.

Supplementary Note 8: Holliday Junction - sample

A label scheme was chosen to allow resolving the two stacked conformations (HF: high FRET and MF: middle FRET) and the potentially planar conformations (LF: low FRET) of the Holliday Junction (HJ). For the computation of the expected FRET efficiencies, we used in-house software based on FRET positioning screening [10]. The measured distances from Photon Distribution Analysis (PDA) for the HF and MF states were slightly higher than expected (see Supplementary Table 1). For the calculation a Förster Radius of $R_0 = 49.3$ Å was used. The observed shift of around 6 Å towards higher distances implies a distance of around 60 Å for the LF planar state of the HJ, leading to roughly $E = 0.2$. The labeling quality was monitored using stoichiometry and anisotropy. As one can see in Supplementary Fig. 9**a**, most of the molecules were doubly labeled, and only minor fractions of donor- and acceptor-only molecules appeared. The anisotropy decay was fitted using a two-component Perrin equation [11],

$$r_D(\tau_{D(A)}) = r_0 \left[\frac{X}{1 + \frac{\tau_{D(A)}}{\rho_1}} - \frac{1-X}{1 + \frac{\tau_{D(A)}}{\rho_2}} \right], \tag{11}$$

where the quantities ρ_1 and ρ_2 denote the characteristic rotational time of the dye and the HJ, respectively, and X corresponds to the fraction of the species rotating with a characteristic rotational time ρ_1. As expected, the anisotropy decay is mostly described by the rotation of the dye (X=0.87).

FRET level (state)	predicted $< R_{DA} >$[Å]	$< R_{DA} >$[Å] with OFA	$< R_{DA} >$[Å] without OFA
HF (stacked HJ)	36	38.4	39
MF (stacked HJ)	46	51.5	52.7
LF (planar HJ)	54	expected: ≈ 60	expected: ≈ 60

Supplementary Table 1: Comparison of the calculated distances using FPS to the in PDA measured distances for the measurement with 1 mM Mg^{2+}.

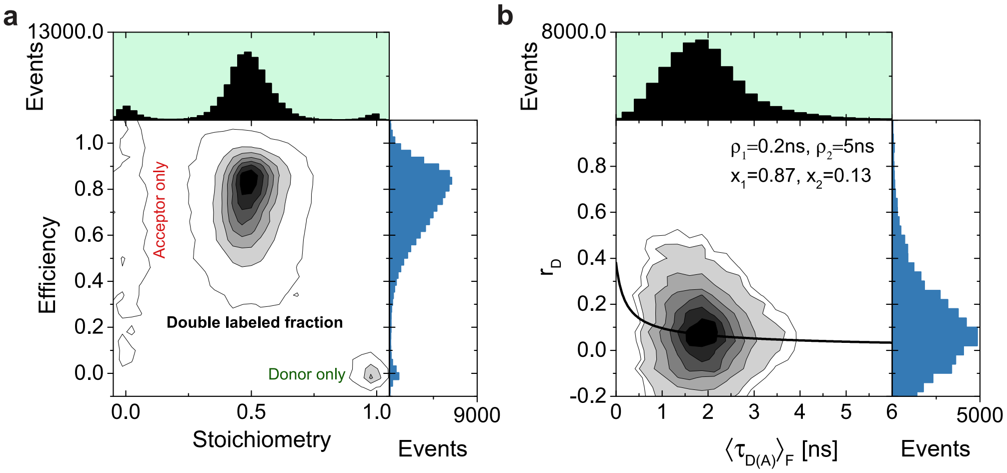

Supplementary Fig. 9: **Monitoring the hybridization and labeling quality of the HJ. a,** MFD-histogram of stoichiometry and FRET efficiency shows three populations, two minor (acceptor only and donor only) and one major (doubly labeled) populations of HJ. **b,** Lifetime anisotropy diagram with an overlapped two-component Perrin equation (solid black line). Here, one main population of the doubly labeled HJ is obtained.

Supplementary Note 9: Holliday Junction - Photon Distribution Analysis (PDA)

In order to test the effect of the prolonged diffusion time τ_d on the PDA, simulations similar to those in Ref. [12] were performed. We generated data for a two-state system with FRET efficiencies equal to the ones measured and listed in Supplementary Table 1. We note that in contrast to the real data, we assumed that all molecules show dynamical averaging in the simulations. Supplementary Fig. 10 shows the resulting distribution of burst duration with and without the OFA for the experimental data (**a**) and for the simulated data (**b**). The simulation can reproduce the distributions for the measurements qualitatively with and without the OFA. We note that in the simulations, the diffusion time without the OFA is set to $\tau_d = 0.7$ ms and $\tau_d = 2$ ms in the case of the OFA. Supplementary Fig. 10**c** shows the fraction of molecules with conformational dynamics as a function of the diffusion time. The percentage of active molecules detected with PDA is lower for short diffusion times than for longer detection times in a global analysis of time windows (TWs) with widths of 1, 2 and 3 ms. Moreover, the simulation results show that a factor of 3 in the diffusion time results in a 10 % improvement in detecting dynamic molecules. Furthermore, in the case of molecules with different brightnesses, a difference in the number of dynamic molecules is noticeable for short diffusion times (see Supplementary Fig. 10**d**). The simulated PDA without and with OFA is shown in Supplementary Figs. 10**e** and **f**. Here, it is evident that molecules with longer diffusion time exhibit a higher fraction of dynamic molecules relative to the stable FRET states. Thus, the simulations indicate that the enhanced detection of dynamic molecules is mainly due to the longer observation times, which can explain the observations made in Fig. 3 of the main manuscript.

Supplementary Note 10: HJ - FRET efficiency trajectories

The aim of the measurements using the OFA is to resolve the FRET levels of the molecule on a time scale where the states of the HJ are stable. In this regime, dynamical averaging can be avoided. Thus, we can follow the switching between the FRET levels and, therefore, the transition of the HJ to another state in "real time". However, the poor photon detection of standard optical configurations often implies long integration times when using MFD. For example, in burstwise-based analysis, the signal is integrated over the whole burst duration. Thus, when the exchange rate between the molecule's conformations is faster than the diffusion time, the analysis averages out the FRET efficiency levels into one population. An approach that is not limited to the burst duration is PDA, which is based on equally-sized time windows with a global analysis over different TWs, typically 1, 2 and 3 ms. However, if enough photons are collected, one can also look at the MFD analysis using time windows. Supplementary Fig. 11 shows a comparison between a burstwise based analysis and a time window based analysis for measurements with the OFA. It is clear that the burstwise analysis shows a FRET population mainly off the static FRET line. In contrast, the FRET population is mainly on the static FRET line in the time window-based analysis. This means that dynamical averaging can be avoided at binning times equal to or lower than 200 μs.

We took this idea further to study FRET efficiency trajectories. Supplementary Fig. 12 shows two such measurements at different Mg^{2+} concentrations. Supplementary Figs. 12 **a** and 12 **e** show FRET efficiency trajectories assembled from 70 individual bursts using 0.2 and 0.5 mM Mg^{2+}, respectively. Moreover, the FRET efficiency trajectories were analyzed with an HMM where only two states were sufficient to fit the experimental model appropriately. Supplementary Fig. 12 **b** shows a PDA with TWs of the same size as the time binning (50 μs). It can be seen that the HF state is most occupied. This is decreased as more Mg^{2+} is added (see Supplementary Fig. 12 **f**). The exponential decay of the level duration of the HF and MF states shows a longer relaxation time in the presence of more Mg^{2+}, as expected. This can be seen by comparing Supplementary Figs. 12 **c** and 12**g** or 12**d** and 12**h**. These observations indicate that with the OFA, it is possible to resolve FRET efficiency levels and their trajectory from single-molecule bursts of freely diffusing biomolecules.

To analyze the noise using different time binning of the FRET efficiency trajectory, we followed two approaches. The first is based on the standard deviation of the FRET efficiency, $\sigma(E)$, fitted to the signal by the hidden Markov model (HMM), which was analyzed using the software Hammy [13] (see Supplementary Fig. 13 **a**). The second method for determining $\sigma(E)$ is based on a statistical noise analysis of the total mean

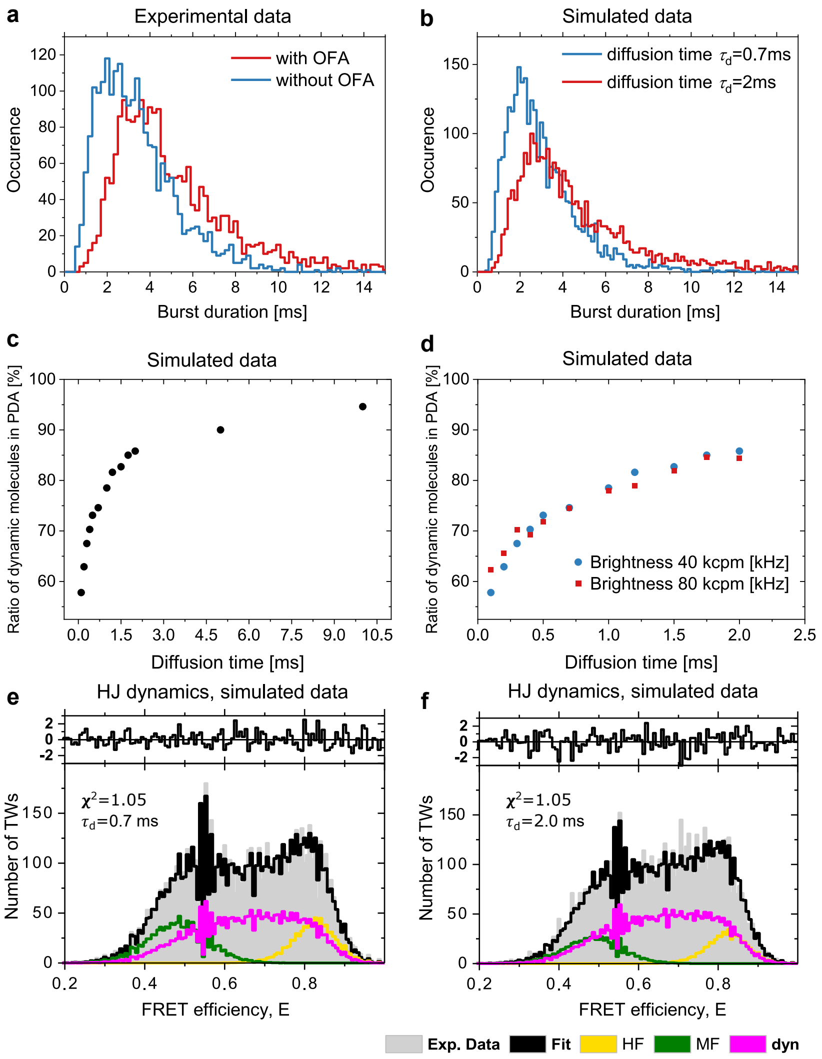

Supplementary Fig. 10: **Simulation of a PDA analysis using a two-state system with different diffusion times. a,** Burst duration distribution obtained from the experimental data for the measurement with and without OFA. **b,** Simulated distribution using a diffusion time of 0.7 ms for the case without OFA and 2 ms for measuring with OFA. **c,** Fraction of dynamic molecules found in the PDA analysis from a simulation using only dynamic molecules as a function of the diffusion time. **d,** Same as **c** but with molecules having different brightness. PDA analysis of the simulated two-state system at fast (**e**) and long (**f**) diffusion times. The distribution of the static HF state is shown in yellow, the distribution of the static MF state is shown in dark green, and the dynamic distribution is shown in magenta. The analysis was done using a global fit with TWs of 1, 2 and 3 ms.

number of photons that were obtained using different time bins. To do so, the following equation was applied

$$\sigma(E) = \sqrt{\frac{1}{N_f}E\left[E\left(1-\frac{1}{\gamma}\right)+\frac{1}{\gamma}\right]}\sqrt{\frac{1-E}{E}}, \tag{12}$$

where N_F is the total mean number of donor and acceptor photons for every time resolution, E is the FRET efficiency and $1/\gamma$ is the inverse normalized photon collection yield with $1/\gamma = (\Psi_D\Phi_{F,D(0)})/(\Psi_A\Phi_{F,A})$ which is based on the experimental detection efficiencies of the donor and acceptor ($\Psi_D = 0.8$, $\Psi_A = 1$), and their effective fluorescence quantum yields ($\Phi_{F,D(0)} = 0.8$, $\Phi_{F,A} = 0.23$) (see Supplementary Fig. 13 **b**).

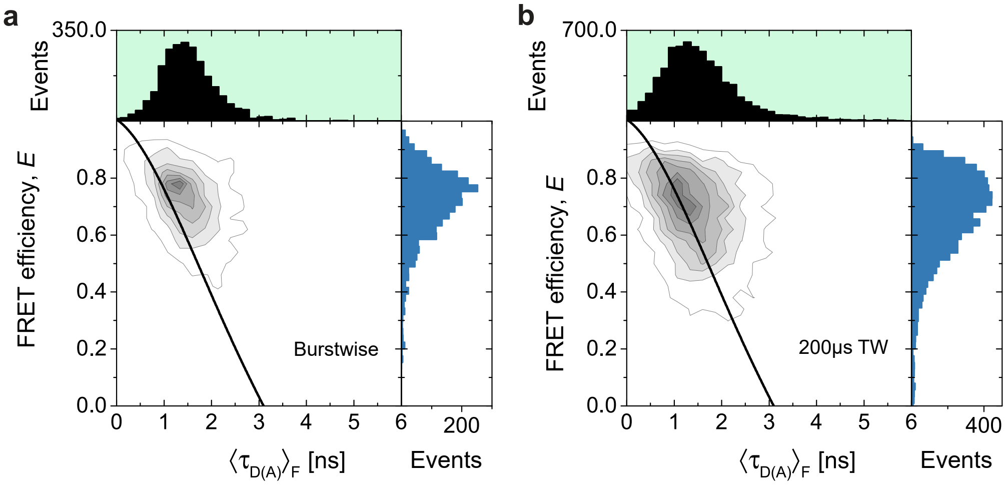

Supplementary Fig. 11: **Comparison of burstwise and time-window based analysis of the HJ in MFD. a,** Burstwise analysis of the sm-MFD measurement of the HJ labeled with Alexa488/Atto647N for a buffer without addition of Mg^{2+}. 2-D histogram of the lifetime of the donor in presence of an acceptor $\langle\tau_{D(A)}\rangle_F$ and the FRET efficiency E. The static FRET line was estimated using $E(\langle\tau_{D(A)}\rangle_F) = 1 - ((0.0126\langle\tau_{D(A)}\rangle_F^4 - 0.148\langle\tau_{D(A)}\rangle_F^3 + 0.5521\langle\tau_{D(A)}\rangle_F^2 + 0.3416\langle\tau_{D(A)}\rangle_F - 0.0174)/3.1)$. The FRET population is clearly shifted off the static FRET line towards a longer lifetime. **b,** TW analysis of the same bursts selected in the burstwise approach (see **a**). Here, every burst is cut into equally 200 μs long TW. The 1D projections show a broadening of the distribution and the TW-bursts no longer are shifted away from the static FRET line. The excitation power used in the experiment is $I_{\mathrm{exc},485} = 800\,\mu\mathrm{W}$.

In equation 13, we define a signal-to-noise ratio (SNR) of a FRET level as

$$SNR = \frac{\Delta E}{\sigma(E_1) + \sigma(E_2)} = \frac{\Delta E}{\sigma_{total}(E_1, E_2)}, \tag{13}$$

where SNR = 1 corresponds to the threshold for resolving two distinct FRET efficiency levels. For SNR-values > 1, the experimental noise is small enough to resolve the two FRET efficiency levels for a given FRET contrast $\Delta E = E_2 - E_1$. Using our measured FRET efficiency levels (E_2 =0.81 and E_1 =0.35 , i.e. $\Delta E = 0.46$, the resulting SNR computed by both methods are depicted in Supplementary Fig 13 **c**. In Supplementary Fig. 13 **d**, the SNR values with and without OFA are compared. It can be seen that with OFA we achieve a time resolution of $\sim 20\mu$s, whereas the observation times without OFA are much longer ($\sim 90\mu$s), even though our FRET contrast of ΔE =0.46 is very high. In conclusion, under our experimental conditions, the temporal resolution is increased by a factor ~ 4.5 in an OFA.

To further test and validate this approach, data were generated for high excitation rates by setting the brightness of the molecules to $Q = 500$ kcpm (kilo counts per molecule) in the case with OFA (see Supplementary Fig. 14 **a**), and $Q = 150$ kcpm without OFA (**b**). The relaxation times of the levels were obtained experimentally (see Supplementary Fig. 12). The HMM algorithm fails to find a reasonable amount of HF states in case of no OFA due to an insufficient number of photons. A close-up of the simulated signal obtained with the OFA is shown in (**c**). Here, it is evident that the FRET efficiency levels are identifiable "by eye". Thus, they are easily fitted with the HMM algorithm, whereas, without the OFA, the FRET efficiency is too noisy for the HMM algorithm to converge (see Fig. 14**d**). The analysis using HMM finds a distribution width of $\sigma_{\mathrm{efficiency}} = 0.14$ in the case of an OFA and $\sigma_{\mathrm{efficiency}} = 0.32$ in open solution.

Supplementary Note 11: Multiparameter Fluorescence Detection (MFD)

Single-molecule Multiparameter Fluorescence Detection (sm-MFD) experiments were performed on a home-built setup based on an Olympus IX70 inverted microscope as described in Ref. [7]. For excitation and collection of the fluorescence signal, we used an Olympus 100x/1.4 NA objective. For excitation in the PIE configuration green (λ_{exc} = 485 nm) and red (λ_{exc} = 640 nm) linearly polarized pulsed diode lasers were used (LDH-D-C 485, PicoQuant and LDH-D-C 640, Picoquant). Both lasers were operated at a repetition rate of 32 MHz [8, 9]. The fluorescence signal was filtered from the excitation light using a triple band beamsplitter 488/570/640 (AHF Analysentechnik). A polarizing beam splitter in the detection path splits the beam into perpendicular and parallel polarizations. Furthermore, the signal from the donor and acceptor molecules in each polarization channel was separated with a dichroic beamsplitter. A further step of filtering using bandpass filters in the donor (ET535/50, AHF Analysentechnik) and acceptor (HQ 730/140, AHF Analysentechnik) channels was taken to minimize the spectral overlap. Finally, each beam was directed to avalanche photodiodes (APD) for detection. Single-photon counting was done with synchronized channels (HydraHarp 400, PicoQuant, Germany) operating in Time-Tagged Time-Resolved (TTTR) mode. Data analysis was performed using in-house software available upon request on the website of the Seidel group (https://www.mpc.hhu.de/software.html). An emCCD camera (DV887 ECS-BV, Andor) was used to position the center of the micropipette above the focus of the laser beam while it was illuminated with a flashlight. The objective was mounted on a closed-loop piezo to ensure high precision and stability of the observation volume (PI, E-662).

Supplementary Note 12: Monte-Carlo simulations of the molecular diffusion

We employed Monte-Carlo simulations to investigate how the antenna geometry modifies the diffusion behavior of single molecules. We assume that the excitation power is low enough to prevent bleaching of the fluorophore. Supplementary Fig. 16 **a** shows a sketch of the structure used to simulate the antenna geometry. Here, we used experimental values for the dimensions of the micropipette. The shape of the water meniscus (water-air interface) was taken to follow an elliptical function with radii of 7 μm in the x and y directions and a radius of 0.5 μm in the z direction. To simulate the observation volume, we defined an elliptical region in the center of the OFA with lateral radius of 0.5 μm and axial radius of 2 μm. When an analyte comes within 10 nm of the simulated volume boundary, the simulation implements a reflection in a random direction. A burst is created every time the analyte enters and exits the observation volume. We used the function '*normrnd*()' from **Matlab** to generate random vectors with three spatial coordinates for simulating the trajectories. We used a time step of 80 ns and a diffusion coefficient of $D = 400\,\mu\text{m}^2/\text{s}$ for the simulations with and without the OFA. Moreover, we considered $N = 10^7$ steps of diffusion which is equal to 0.8 s of diffusion, and included an average of 10^4 trajectories in each simulation. Supplementary Fig. 16 **b** corresponds to the simulation in open solution where no boundaries are imposed.

The results of the simulations reveal a larger number of bursts per unit time with the OFA compared to the case of diffusion in an open solution. Supplementary Fig. 16 **c** shows exemplary time traces obtained with the OFA (red trace) and in an open solution (blue trace). Here, each trace has been normalized to the maximum number of photons collected per burst. The difference in the number and amplitude of bursts in the two scenarios is evident. Furthermore, the dependence of the enhancement in the number of bursts obtained with the OFA as a function of the thickness of the water channel is shown in Supplementary Fig. 16 **d**. Here we can see that as the water channel increases, the number of bursts approaches the case of diffusion in open solution.

To obtain insight into the experimental observation that the water-air interface slows down diffusion, we implemented a potential $\sigma(z)$ to slow down the motion of analytes at this interface (see Supplementary Fig. 16 **e**). The strength of the potential is tuned by the time t_σ that the molecule stays in the region delimited by d_σ. Supplementary Fig. 16 **f** shows the correlation analysis from the simulated trajectories for three different values of t_σ over an extension of the potential in the z-direction given by $d_\sigma = 10$ nm. We

find that in the case of the dye molecule Rhodamine-110, a value of $t_\sigma = 40\,\mu$s can qualitatively reproduce the experimental observations.

We found that the details of the mathematical expression for the potential are not critical. For example, a step-wise function that freezes the motion of the analytes completely or a smooth function such as exponential decay of the analytes' diffusion coefficient conduce to similar results as long as the confinement time is fixed. We emphasize that the idea of the simulations is not to explain the physical mechanism behind the slow-down motion of the analytes but to provide a consistent explanation for the interface processes, which need further research to reach a quantitative understanding.

Supplementary Note 13: Holliday Junction - sm-MFD

Supplementary Figs. 17**a** and **b** show the comparison of the HJ dynamics in an open liquid (purple) and inside the optofluidic antenna (yellow) for a buffer solution without the addition of Mg^{2+} (**a**) and for a buffer solution at 1 mM Mg^{2+} concentration (**b**). We observe no change in the dynamics of HJ for diffusion inside the OFA compared to the case of the open solution.

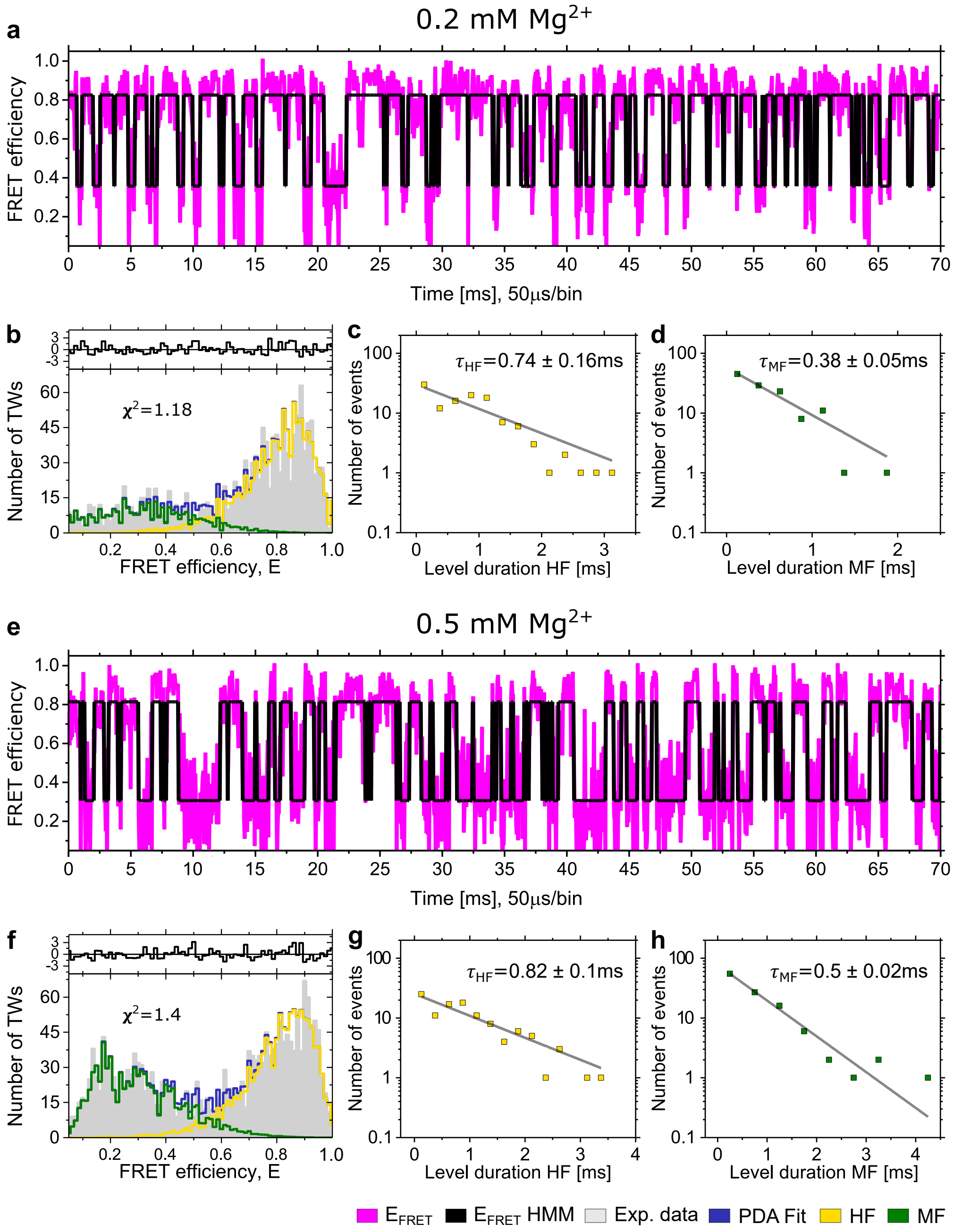

Supplementary Fig. 12: **Holliday Junction FRET Efficiency trajectory analysis at different Mg^{2+} concentrations. a,** FRET efficiency trajectory consisting of single bursts measured in a buffer at 0.2 mM Mg^{2+} concentration. The efficiency levels (black curve) were fitted using a HMM algorithm. **b,** Histogram of the trajectory shown in **a** which has been divided into time windows with a binning size of 50 μs. The resulting histogram is then fitted using PDA. The dark-green distribution shows the MF-state of the HJ and the yellow distribution shows the HF-state of the HJ. The Supplementary Figs. **c** and **d** show the distribution of the level durations of the HF-state (**c**) and the MF-state (**d**) in a semi logarithmic plot. The distribution was fitted using an exponential decay without an offset (gray curve). **e,** FRET efficiency trajectory consisting of single bursts measured in a buffer (0.5 mM Mg^{2+} concentration). **f,** Resulting histogram from **e** with PDA using time windows with 50 μs binning time. The Supplementary Figs. **g** and **h** show the distribution of the level durations of the HF-state (**g**) and the MF-state (**h**) in a semi logarithmic plot.

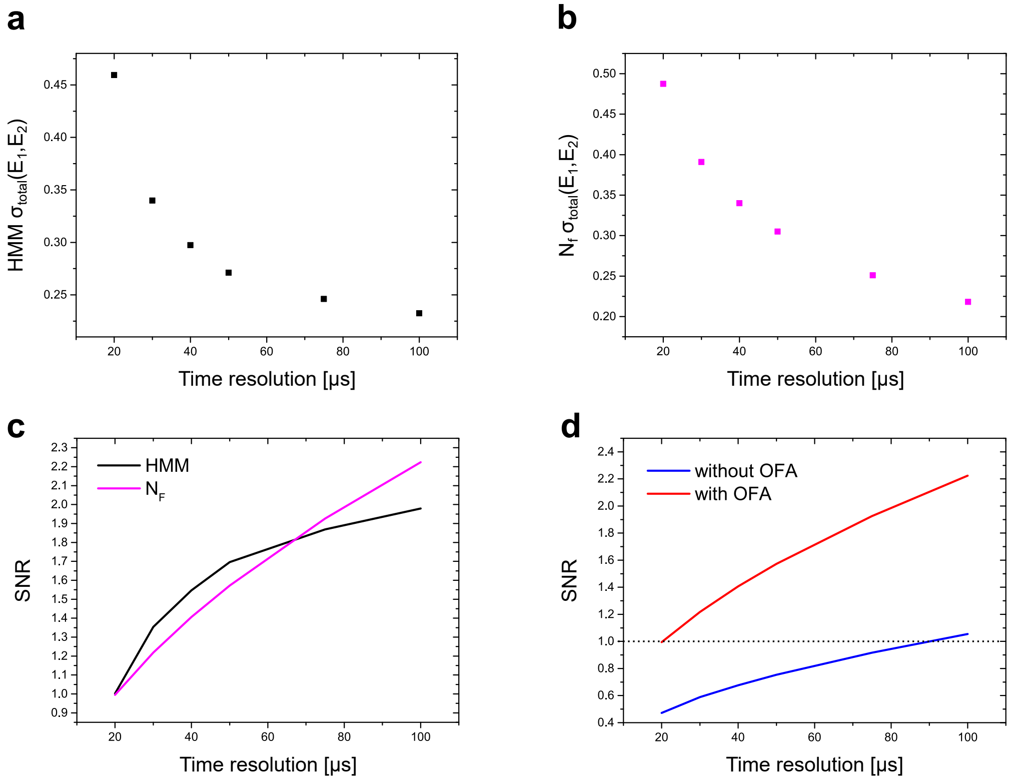

Supplementary Fig. 13: **Noise analysis of a FRET efficiency trajectory of the HJ. a,** Standard deviation of the FRET efficiency for the two distinct FRET states of the HJ in dependence on the time resolution obtained by the Hidden-Markov Model. **b,** Standard deviation of the FRET efficiency for the two distinct FRET states of the HJ as a function of the mean number of photons N_f contained in individual bursts. **c,** Resulting SNR for the HMM (black line) and the mean number of photons N_F (magenta line) using eq.12 and 13. **d,** Difference in the SNR using the OFA (red) and not using the OFA (blue). The black dotted horizontal line indicates the SNR threshold of 1.

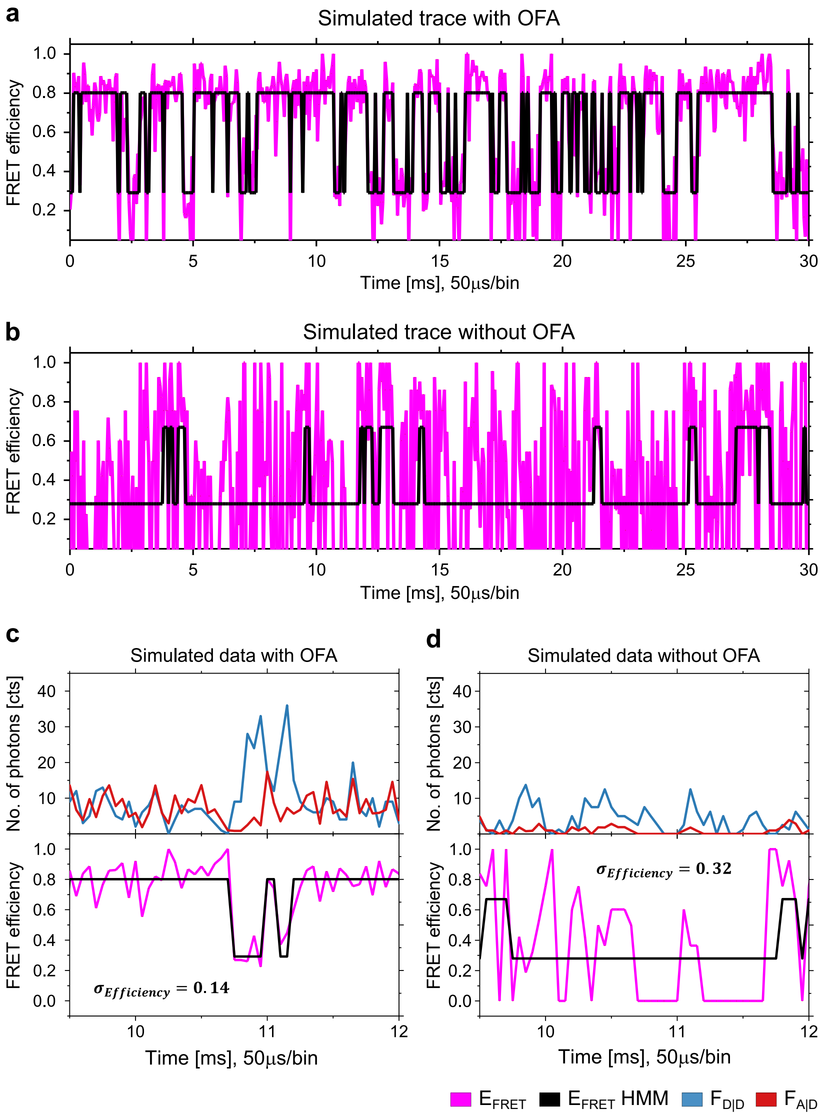

Supplementary Fig. 14: **Simulated FRET Efficiency trajectories with and without OFA. a,** FRET efficiency trajectory simulated considering the rates obtained from measurements at 0.2 mM Mg^{2+} concentration. In the simulation we use a two state model for the HF and MF states of the HJ. Sm-bursts were filtered for a minimum burst duration of 0.8 ms and merged to a single trajectory lasting around 70 ms. FRET levels were fitted using a HMM algorithm. **b,** The same simulation as in **a** was repeated using molecules with 1/4 of the brightness to mimic the case of no OFA. FRET efficiency was fitted again using a HMM algorithm. Supplementary Figs. **c** and **d** show a close-up in the FRET efficiency trajectory with the OFA (**c**) and without the OFA (**d**). The top panel in both plots show the FRET efficiency trajectory of the fluorescence for the donor under donor excitation $F_{D|D}$ and the fluorescence of the acceptor under donor excitation $F_{A|D}$. The resulting $\sigma_{\text{efficiency}}$ obtained from the HMM-fit is indicated in each plot.

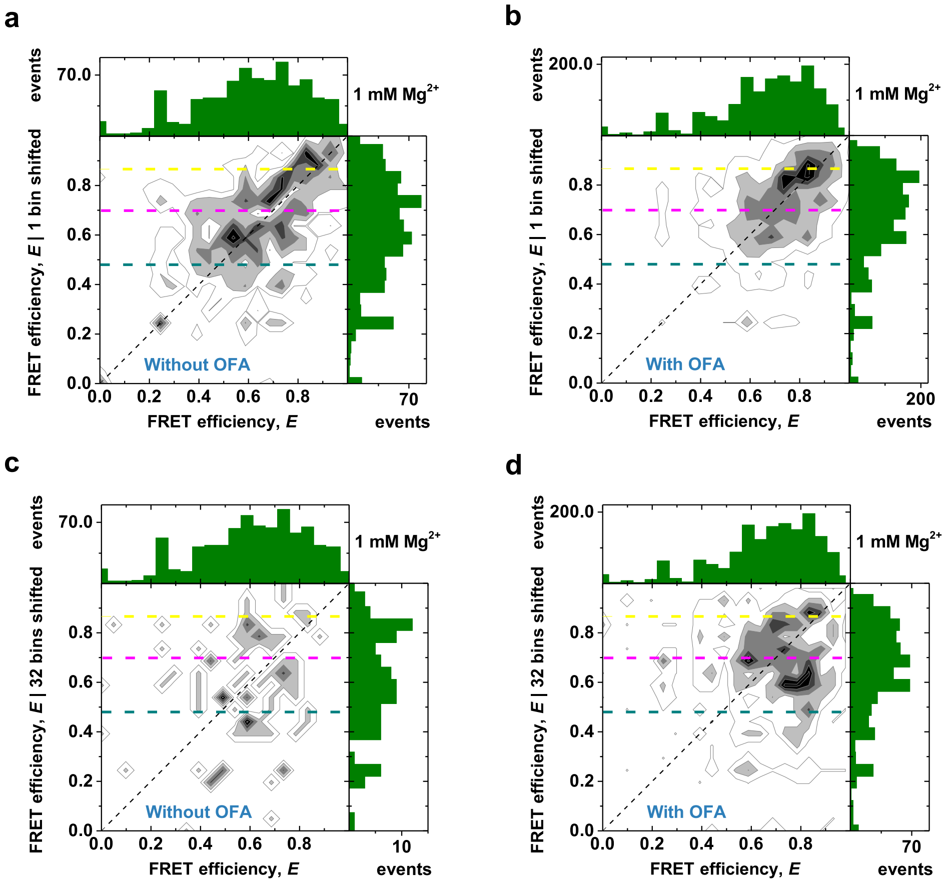

Supplementary Fig. 15: **Recurrence E contour plots of the HJ at 1 mM MgCl2 for measurements without and with OFA, respectively.** The FRET efficiency (E) trajectories within a burst were binned in time windows of 0.3 ms. The FRET efficiency E of the start bin is compared to the E-value of a second time window that is shifted by the given number of time bins of 0.3 ms. For the shift by one time bin of 0.3 ms the number of recurrence events (y-axis) without (**a**) and with OFA (**b**) does not differ significantly. However, due to the higher mean number of detected photons, N_F, with the OFA, the shot noise and the corresponding width of the FRET efficiency histograms are lower. Most events lie on the diagonal that indicate the transition to other FRET efficiencies has taken place. At larger time shifts of 32 bins (i.e. 6.6 ms), the difference between measurements without (**c**) and with (**d**) OFA is obvious. Comparing both conditions, the number of events with OFA dropped to 1/7 (**c**) in comparison to 1/3 (**d**) with OFA. Moreover, most events are off-diagonal events, which indicates that transitions to distinct FRET efficiency species took place. This demonstrates that measurements with OFA have more recurrence events and lower shot noise, so that the time window for the analysis of very slow exchange kinetics is expanded.

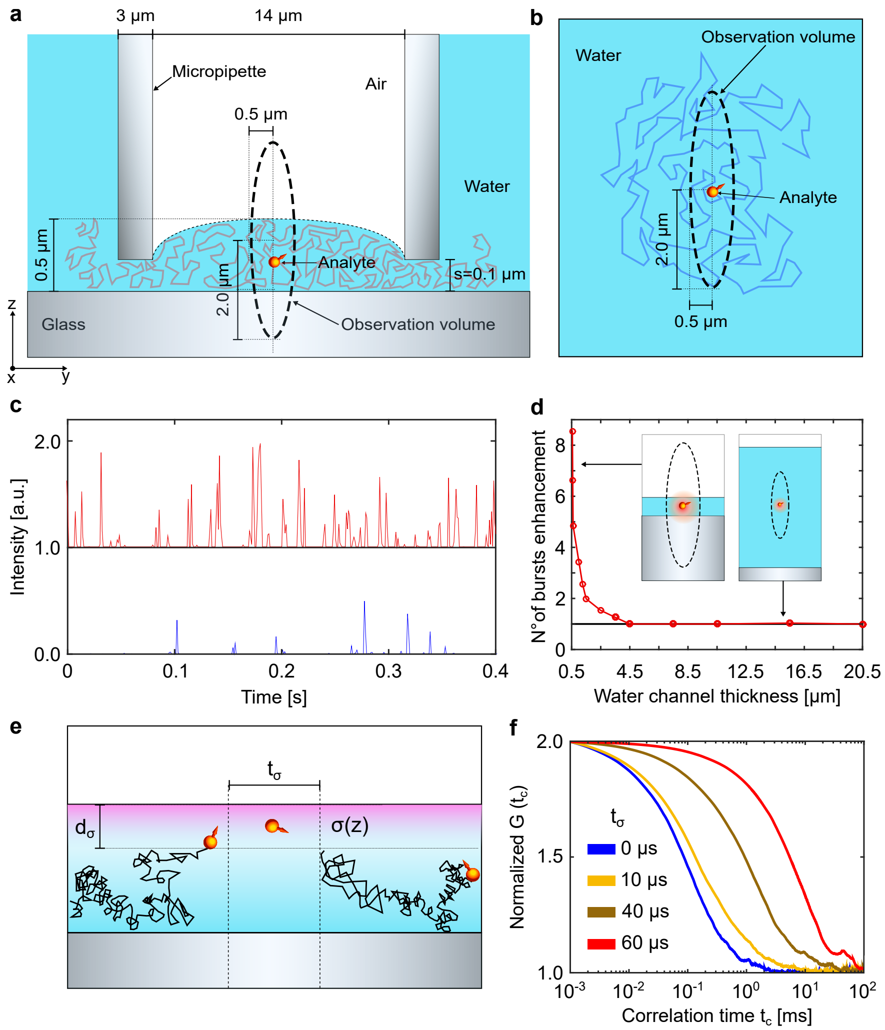

Supplementary Fig. 16: **Diffusion inside the OFA: Monte-Carlo simulation.** Sketch of the simulation where a single analyte diffuses inside the OFA (**a**) and in bulk solution (**b**). The red and blue trajectories illustrate a possible path of the analytes inside the OFA and in bulk, respectively. **c,** Exemplary time traces simulated inside the OFA (red trace) and in bulk solution (blue trace). **d,** Enhancement in the number of bursts obtained with the OFA relative to the case of diffusion in an open solution. The enhancement in the number of bursts is shown for different thicknesses of the water channel. Insert: illustration of two extreme thicknesses of the water layer. **e,** Sketch of the simulation where a potential $\sigma(z)$ (pink region) is defined at the water-air interface. The potential extends along the complete interface in the x-y-directions. Moreover, the extension of the potential in the z-direction is given by $d_\sigma = 10\,\text{nm}$. The strength of the potential is modulated by the time t_σ that the molecules spend in the region defined by d_σ. **f,** Correlation analysis of the simulated time traces for different values of t_σ and a fix value of $d_\sigma = 10\,\text{nm}$.

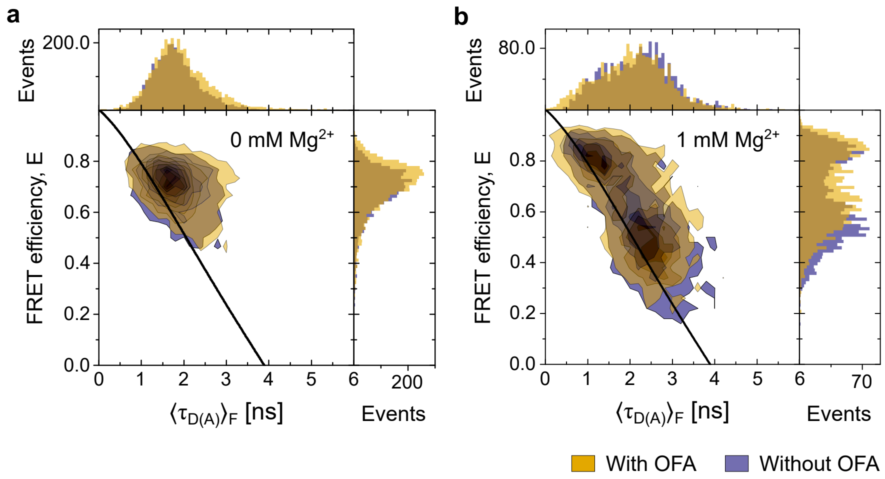

Supplementary Fig. 17: **Holliday junction single molecule multiparameter fluorescence detection (sm-MFD): Comparison between measurements with and without using the OFA. a,** Two dimensional histogram of the donor fluorescence lifetime in presence of an acceptor $\left\langle\tau_{D(A)}\right\rangle_F$ and the FRET efficiency E. The black line (static FRET line) describes the relation of intensity and donor lifetime based equal FRET efficiencies and bursts from molecules with a single conformation state are distributed on this line. The molecules show a fast dynamic between their conformational states and therefore a high averaging of the FRET efficiency resulting in only one visible population. The plot in purple corresponds to the case of an open solution without OFA. **b,** Adding 1 mM Mg^{2+} slows down the dynamical behavior and two populations. This effect become visible in both cases.

References

[1] Felekyan, S., Kalinin, S., Sanabria, H., Valeri, A. & Seidel, C. A. M. Filtered FCS: species auto- and cross-correlation functions highlight binding and dynamics in biomolecules. *ChemPhysChem* **13**, 1036–1053 (2012).

[2] Rigler, R., Mets, U., Widengren, J. & Kask, P. Fluorescence correlation spectroscopy with high count rate and low background: analysis of translational diffusion. *Eur. Biophys. J.* **22** (1993).

[3] Basché, T., Moerner, W. E., Orrit, M. & Wild, U. P. *Single-Molecule Optical Detection, Imaging and Spectroscopy* (Wiley-VCH Verlag GmbH & Co. KGaA, Weinheim, Germany, 2008).

[4] Novotny, L. & Hecht., B. *Principles of Nano-Optics.* (Cambridge University Press, New York, United States, 2006).

[5] Qian, H. & Elson, E. L. Analysis of confocal laser-microscope optics for 3-D fluorescence correlation spectroscopy. *Appl. Opt.* **30**, 1185–1195 (1991).

[6] Mortensen, K. I., Churchman, L. S., Spudich, J. A. & Flyvbjerg, H. Optimized localization analysis for single-molecule tracking and super-resolution microscopy. *Nat. Methods.* **7**, 377–381 (2010).

[7] Widengren, J. Single-molecule detection and identification of multiple species by multiparameter fluorescence detection. *Anal. Chem.* **78**, 2039–2050 (2006).

[8] Kudryavtsev, V. *et al.* Combining MFD and PIE for accurate single-pair Förster resonance energy transfer measurements. *ChemPhysChem* **13**, 1060–1078 (2012).

[9] Hendrix, J. & Lamb, D. C. Pulsed interleaved excitation. *Meth. Enzymol.* 205–243 (2013).

[10] Kalinin, S. *et al.* A toolkit and benchmark study for FRET-restrained high-precision structural modeling. *Nat. Methods* **9**, 1218–1225 (2012).

[11] Lakowicz, J. R. *Principles of fluorescence spectroscopy* (Springer, New York, United States, 2006).

[12] Kalinin, S., Valeri, A., Antonik, M., Felekyan, S. & Seidel, C. A. M. Detection of structural dynamics by FRET: a photon distribution and fluorescence lifetime analysis of systems with multiple states. *J. Phys. Chem. B* **114**, 7983–7995 (2010).

[13] McKinney, S. A., Joo, C. & Ha, T. Analysis of single-molecule FRET trajectories using hidden Markov modeling. *Biophys. J.* **91**, 1941–1951 (2006).
